# Prevalence and Associated Factors for Depressive Symptomatology in Chinese Adults During COVID-19 Epidemic

**DOI:** 10.3389/fpsyg.2020.616723

**Published:** 2020-12-23

**Authors:** Songxu Peng, Xin Lai, Yukai Du, Yuting Li, Kunming Tian, Yong Gan

**Affiliations:** ^1^Department of Maternal and Child Health, Xiangya School of Public Health, Central South University, Changsha, China; ^2^Department of Maternal and Child Health, School of Public Health, Tongji Medical College, Huazhong University of Science and Technology, Wuhan, China; ^3^Cancer Center, Union Hospital, Tongji Medical College, Huazhong University of Science and Technology, Wuhan, China; ^4^Department of Preventive Medicine, School of Public Health, Zunyi Medical University, Zunyi, China; ^5^Institute of Reproductive Health, Tongji Medical College, Huazhong University of Science and Technology, Wuhan, China; ^6^Department of Social Medicine and Health Management, School of Public Health, Tongji Medical College, Huazhong University of Science and Technology, Wuhan, China

**Keywords:** epidemic, COVID-19, risk factors, prevalence, depression symptoms

## Abstract

**Background:** The coronavirus disease 2019 (COVID-19) has been rapidly transmitted worldwide, which contributed to various psychological problems (such as fear, depression, and anxiety) among the general population in China. The purpose of this study is to investigate the prevalence and associated factors of depressive symptoms among Chinese adults.

**Methods:** A cross-sectional study of Chinese adults was conducted during 17–29 February 2020. Symptoms of depression were assessed using the Center for Epidemiologic Studies Depression scale (CES-D).

**Results:** A total of 3,399 respondents were included in the analysis. It was observed that 14.2% (481/3,399) of the participants were screened positive for depressive symptoms. In a multivariate logistic regression analysis, older age (OR = 0.98; 95% CI, 0.97–0.99), smoking (OR = 1.57; 95% CI, 1.10–2.26), self-rated health (good: OR = 0.49; 95% CI, 0.37–0.66; fairly: OR = 0.60; 95% CI, 0.45–0.80), having greater support scores (OR = 0.95; 95% CI, 0.94–0.96), knowledge about the main symptom of COVID-19 (very clearly: OR = 0.58; 95% CI, 0.42–0.79; relatively clearly: OR = 0.59; 95% CI, 0.44–0.79), and staying in Wuhan within 3 months before the outbreak of epidemic (OR = 1.78; 95% CI, 1.34–2.38) were associated with depressive symptoms.

**Conclusion:** A considerable proportion of the general population in China had depressive symptoms during the COVID-19 epidemic. Routine screening and targeted interventions for depression are needed among high-risk depressed individuals during the COVID-19 epidemic.

## Background

The coronavirus disease 2019 (COVID-19) spread rapidly worldwide, causing high morbidity and heavy economic burden (Docherty et al., 2020; Karagiannidis et al., 2020; Onder et al., 2020; Tadesse et al., 2020; Tang et al., 2020; Wu and McGoogan, 2020; Loomba et al., 2021). In December 2019, a pneumonia of unknown cause was detected and became an epidemic in Wuhan, China. On 7 January 2020, the severe acute respiratory syndrome coronavirus 2 (SARS-CoV-2) was identified as the etiological agent of the epidemic by genome sequencing (Zhu et al., 2020). Because of the quick spreading of the virus in multiple countries, at the meeting of the emergency committee of the World Health Organization (WHO) on 30 January 2020, COVID-19 was declared a Public Health Emergency of International Concern (PHEIC) (World Health Organization, 2020a). Subsequently, WHO formally named COVID-19 on 11 February 2020 (World Health Organization, 2020b). According to the official website of the National Health Commission, on 17 February 2020, a total of 72,436 confirmed cases and 1,868 death cases were identified in China, with a fatality rate of 2.6% (The National Health Commission of China, 2020). The COVID-19 outbreak posed great threat to human life (Wang et al., 2020a). Additionally, findings from previous studies suggested that outbreaks of major communicable diseases, including severe acute respiratory syndrome and Middle East respiratory syndrome, increased the risk of depression in the populations affected (Mak et al., 2009; Keita et al., 2017). Compared with previous epidemics and pandemics, COVID-19 is more contagious and spreads faster (Meo et al., 2020), which may further aggravate the public’s depression. Therefore, timely psychological assessment and appropriate intervention to prevent depressive symptom is necessary.

Depression is a common mood disorder characterized by persistently low mood state, affecting 1.4–37.5% of the population (Maske et al., 2016; Rotenstein et al., 2016; Arias-de la Torre et al., 2018). Several studies had shown that depression not only reduces the quality of life of patients but also increases the risk of chronic physical diseases and suicide (Whooley and Wong, 2013; Kozela et al., 2016; Lopez et al., 2018). Recent studies have shown that the prevalence of depressive symptoms in the general population is high during the COVID-19 epidemic, ranging from 3.7 to 68% (Ettman et al., 2020; Goularte et al., 2020; Rodríguez-Rey et al., 2020; Tan et al., 2020; van der Velden et al., 2020). A published meta-analysis study included 14 studies and suggested that 33.7% of the general population had depressive symptoms during the COVID-19 pandemic (Salari et al., 2020). Additionally, several Chinese studies reported the prevalence of depressive symptoms in the general population during the COVID-19 outbreak. For example, a survey study conducted by Shi et al. (2020) with 56,679 Chinese participants reported 27.9% of the general population had depressive symptoms. A previous study from Shenzhen suggested that the prevalence of depressive symptom was 6.2% in the general population quarantined (Peng et al., 2020), performing in subjects with quarantine experience. Another study by Lei et al. (2020) found the prevalence of depression was approximately 14.6%, with mixed samples including people affected and people unaffected. Therefore, the prevalence of depressive symptoms among the general population is still inconsistent, probably due to the different population and sampling issues in these studies, which deserves further study.

In the past decades, risk factors for depressive symptoms have frequently been examined (Hammen, 2018). However, few studies have explored epidemic-related factors causing depressive symptoms. As a newly emerging disease, the public has limited knowledge about main symptoms, latent period, and route of transmission of COVID-19, and the number of confirmed cases varies from region to region, which may influence the degree of depressive symptoms in the general population. Additionally, Wuhan, as the center of the epidemic in China, whether people with a history of exposure to Wuhan before the COVID-19 epidemic outbreak have a higher risk of depression, it is unclear. To better allocate limited medical resources and formulate effective interventions, the prevalence of depressive symptoms and epidemic-related factors that are associated with them during the COVID-19 outbreak need to be determined urgently.

Therefore, we enrolled a large number of participants and investigated sociodemographic information and epidemic-related factors, which would provide a more complete overview of the risk factors of depressive symptoms. This study aimed to investigate the prevalence and identify the associated factors of depressive symptoms among the general public during COVID-19 epidemic.

## Materials and Methods

### Study Design and Participants

We conducted a cross-sectional online survey to assess the prevalence and risk factors of depressive symptoms among the general population in China during the COVID-19 epidemic. An online survey was used because of the self-isolation of the general population in China. We adopted snowball sampling strategy to recruit participators; potential participators were electronically invited via WeChat. The recruitment information of the study was posted on the university website. Data collection was completed during 17–29 February 2020 period. This survey period corresponded to the reduction stage after the peak point of the COVID-19 epidemic outbreak in the country.

The present study was approved by the Institutional Review Board of Tongji Medical College, Huazhong University of Science and Technology. Informed consent was obtained from the participants before their participation in the study.

### Data Collection

A self-administered questionnaire was used to collect data on sociodemographic characteristics, knowledge about COVID-19, information regarding depressive symptoms and social support, and additional information related to COVID-19. Sociodemographic data included age (continuous variable, ranged from 18 to 99), gender (dummy coded, 0 = female, 1 = male), nationality (dummy coded, 0 = Han, 1 = Other), marital status (dummy coded, 0 = unmarried, 1 = married), employment status (dummy coded, 0 = unemployed, 1 = employed), education (dummy coded, 0 = high school and below, 1 = college, 2 = bachelor or above), type of residence (dummy coded, 0 = urban, 1 = rural, 2 = urban-rural district), smoking (dummy coded, 0 = non-smoker, 1 = smoker) and drinking status (dummy coded, 0 = non-drinking, 1 = drinking), and history of chronic diseases (dummy coded, 0 = no, 1 = yes). Respondents were asked to rate their physical health status (dummy coded, 0 = average/poor, 1 = fairly good, 2 = very good). Knowledge about COVID-19 included the main symptom, latent period, and routes of transmission of COVID-19 (dummy coded, 0 = general, 1 = relatively clear, 2 = very clear). Additionally, respondents were asked to answer if they stayed in Wuhan within 3 months before the epidemic outbreak or had any relatives or friends who were infected with COVID-19 (dummy coded, 0 = no, 1 = yes). Depressive symptoms and social support were assessed by the Center for Epidemiologic Studies Depression scale (CES-D) and Perceived Social Support Scale (PSSS), respectively.

### Social Support

The PSSS was designed by Zimet et al. (1990). The scale has 12 items including three dimensions, namely, family support, friend support, and other support. It is scored on a 7-point Likert-type scale, with higher scores indicating higher perception of social support. The Chinese version of PSSS has been proven to be highly reliable and valid among the Chinese population (Ye et al., 2017). Here, the Cronbach’s alpha value was 0.96.

### Depression Symptoms

The CES-D was used to assess depressive symptoms. It comprises 20 items; each item is rated on a 4-point scale ranging from 0 (“rarely or none of the time”) to 3 (“most or almost all of the time”). The total score ranges from 0 to 60, with a higher score indicating more severe depressive symptoms. For the original CES-D scale, a total score of 16 is used to detect the presence of depressive symptoms (Radloff, 1977). However, a large number of studies have assessed the diagnostic accuracy of the CES-D to detect depression in the general population and proposed a variety of cut-off scores, such as a cut-off score of 18 among elders living in residential homes (Dozeman et al., 2011) and a cut-off score of 22 in older Chinese (Cheng and Chan, 2005). A meta-analytic study systematically reviewed 28 CES-D studies including several Chinese studies and proposed an optimal cut-off score of 20 (Vilagut et al., 2016). Hence, a cut-off value of 20 or a greater total score was considered indicative of depressive symptoms, consistent with the previous research (Jiang et al., 2019). This Chinese version of the CES-D has good reliability and validity and has been widely adopted in the Chinese population. In the present study, the Cronbach’s alpha coefficient for this scale was 0.96.

### Statistical Analysis

Continuous variables were expressed as the mean ± standard deviation (SD) and compared using Student’s *t*-tests. Categorical data were presented as percentages and compared using Chi-square tests. A multivariable logistical regression model was used to identify the risk factors for depression. Statistical significance was assessed at 5% level (two-tailed test). All analyses were performed using SPSS software version 18.0 (SPSS, Chicago, IL, United States).

## Results

### Characteristics of Study Population

A total of 3,405 completed questionnaires were received, and six respondents who lived abroad were excluded from this study. Finally, 3,399 respondents were included in the analysis. The 3,399 respondents from 280 cities in China had an average age of 27.5 years (range, 18–80) with standard deviation of 11.4 years; 95.5% of them were of Han nationality, and 66.5% were female.

Based on the presence of depressive symptoms, all participants were categorized into two groups: the depression and non-depression groups; 14.2% (481/3,399) of the participants were screened positive for depressive symptoms in the present study and included in the depression group. Table 1 compares the sociodemographic and behavior characteristics of participants with and without depressive symptoms. Participants in the depression group were more likely to have a younger age, lower medical insurance coverage, worse self-rated health, and lower support scores compared with those in the non-depression group. In contrast, participants in the non-depression group were likely to be married and employed, female, and non-smoker. However, there was no significant difference in nationality, education, types of residence, alcohol use, and history of chronic disease between the depression and non-depression groups.

**TABLE 1:** Demographic and behavior characteristics of participants with or without depressive symptoms.

Characteristics	Depression (*n* = 481)	Non-depression (*n* = 2,918)	*t/x*^2^	*P*-value
Age (years, mean ± *SD*)	26.3 ± 12.5	27.8 ± 11.2	2.42	0.016
Female (%)	299 (62.1)	1,962(67.2)	4.78	0.029
Han nationality	455 (94.6)	2,794(95.8)	1.31	0.253
Married	121 (25.2)	985 (33.8)	13.91	0.000
Educational level (years)			0.47	0.790
High school and below	55 (11.4)	331 (11.3)		
College	65 (13.5)	429 (14.7)		
Bachelor or above	361 (75.1)	2,158(74.0)		
Type of residence			0.05	0.975
Urban	282 (58.6)	1,695(58.1)		
Rural	154 (32.0)	946 (32.4)		
Urban–rural district	45 (9.4)	277 (9.5)		
Employed	163 (33.9)	1,167(40.0)	6.46	0.011
Smoker (%)	51 (10.6)	226 (7.7)	4.51	0.034
Alcohol (%)	72 (15.0)	398 (13.6)	0.61	0.434
Chronic disease	49 (10.2)	240 (8.2)	2.04	0.153
Medical insurance coverage	398 (82.7)	2,544(87.2)	6.99	0.008
Self-rated health			57.09	0.000
Very good	194 (40.3)	1,527(52.3)		
Quite good	170 (35.3)	1,040(35.6)		
Average	117 (24.3)	351 (12.0)		
Support scores	57.8 ± 13.8	67.4 ± 11.6	14.34	0.000

*SD, standard deviation.*

### Comparison of Concerns and Knowledge About COVID-19 Among Depression and Non-depression Groups

Regarding concerns and knowledge about COVID-19, Table 2 shows that participants in the depression group reported a higher exposure of staying in Wuhan within 3 months before the epidemic outbreak compared with those in the non-depression group. Additionally, participants in the depression group were less knowledgeable about the main symptoms, latent period, and routes of transmission of COVID-19 than those in the non-depression group.

**TABLE 2:** Comparison of concerns and knowledge’s of COVID-19 between people with or without depressive symptoms.

Characteristics	Depression (*n* = 481)	Non-depression (*n* = 2,918)	*x*^2^	*P*-value
**Local cases**			2.14	0.544
< 500	100 (20.8)	542 (18.6)		
500–1,000	271 (56.3)	1,743(59.7)		
1,000–10,000	53 (11.0)	304 (10.4)		
> 10,000	57 (11.9)	329 (11.3)		
**Staying in Wuhan during 3 months before the outbreak of epidemic?**			6.74	0.009
Yes	76 (15.8)	339 (11.6)		
No	405 (84.2)	2,579(88.4)		
**Whether you or your relatives are confirmed by COVID-19?**			2.13	0.144
Yes	5 (1.0)	12 (0.4)		
No	476 (99.0)	2,906(99.6)		
**Do you know the main symptom of COVID-19?**			57.72	0.000
Very clear	134 (27.9)	1,098(37.6)		
Relatively clear	245 (50.9)	1,533(52.5)		
General	102 (21.2)	287 (9.8)		
**Do you know the latent period of COVID-19?**			37.77	0.000
Very clear	185 (38.5)	1,446(49.6)		
Relatively clear	229 (47.6)	1,270(43.5)		
General	367 (13.9)	202 (6.9)		
**Do you know the routes of transmission of COVID-19?**			44.58	0.000
Very clear	181 (37.6)	1,396(47.8)		
Relatively clear	234 (48.6)	1,347(46.2)		
General	66 (13.7)	175 (6.0)		

*COVID-19, 2019 coronavirus disease.*

### Multivariate Regression Analysis in Identifying Risk Factors of Depression

This study used multivariate logistical regression analysis to identify risk factors of depressive symptoms. Variables with statistical significance in univariate analysis, including age, gender, marital status, employment status, smoking status, medical insurance coverage, self-rated health, support scores, staying in Wuhan within 3 months before the epidemic outbreak, and knowledge about the main symptoms, latent period, and routes of transmission of COVID-19, were included in the logistical regression model. Results showed that older age (OR = 0.98; 95% CI, 0.97–0.99; *P* = 0.000), very good self-rated health (OR = 0.49; 95% CI, 0.37–0.66; *P* = 0.000), fairly good self-rated health (OR = 0.60; 95% CI, 0.45–0.80; *P* = 0.001), greater support scores (OR = 0.95; 95% CI, 0.94–0.96; *P* = 0.000), very clear knowledge about the main symptoms of COVID-19 (OR = 0.58; 95% CI, 0.42–0.79; *P* = 0.001), and relatively clear knowledge about the main symptoms of COVID-19 (OR = 0.59; 95% CI, 0.44–0.79; *P* = 0.000) were associated with a decreased risk of depressive symptoms. However, smoking (OR = 1.57; 95% CI, 1.10–2.26; *P* = 0.014) and staying in Wuhan within 3 months before the epidemic outbreak (OR = 1.78; 95% CI, 1.34–2.38; *P* = 0.000) were associated with an increased risk of depressive symptoms (Table 3).

**TABLE 3:** Multivariable logistic analyses for factors related to depressive symptoms.

	Coefficients B	SE	Wald	*P*-value	OR	95% confidence interval for OR	
						Lower	Upper
Age	–0.019	0.005	14.702	0.000	0.981	0.972	0.991
Smoking	0.454	0.184	6.078	0.014	1.574	1.097	2.258
Self-rated health			23.296	0.000			
Very good	–0.7	0.145	23.171	0.000	0.497	0.373	0.660
Fairly good	–0.511	0.147	12.039	0.001	0.600	0.450	0.801
Average/poor					Reference		
Support scores	–0.055	0.004	169.889	0.000	0.947	0.939	0.955
**Do you know the main symptom of COVID-19?**			14.583	0.001			
Very clear	–0.551	0.162	11.528	0.001	0.577	0.42	0.792
Relatively clear	–0.525	0.146	12.96	0.000	0.591	0.444	0.787
General					Reference		
Staying in Wuhan	0.578	0.147	15.382	0.000	1.783	1.335	2.38
(Constant)	2.98	0.309	93.239	0	19.698		

*COVID-19, 2019 coronavirus disease; OR, odds ratio; CI, confidence interval. Included variables: age, gender, married status, employment status, smoking status, medical insurance coverage, self-rated health, social support, staying in Wuhan within 3 months before the outbreak of epidemic, knowledge’s about the main symptoms, latent period, routes of transmission of COVID-19.*

## Discussion

This cross-sectional study, based on 3,399 participants assessed the prevalence and risk factors of depressive symptoms among the Chinese general population during the COVID-19 epidemic. We found that 14.2% of the general population suffered from depressive symptoms during the COVID-19 epidemic. Furthermore, some demographic and concerns as well as knowledge about COVID-19 variables were found to be the influencing factors for depressive symptoms, including age, smoking status, self-rated health, social support, knowledge about the main symptoms of COVID-19, and staying in Wuhan within 3 months before the outbreak.

The prevalence of depressive symptoms among the general population in China during the COVID-19 epidemic in the present study is similar to that in recent published studies. The study by Wang et al. (2020b) with 1,210 respondents from 194 cities in China reported that 16.5% of the general population had moderate to severe depressive symptoms. Lei et al. (2020) reported that the prevalence of depressive symptoms in the public affected and those unaffected was 14.6% during the COVID-19 epidemic. However, a high prevalence of depressive symptoms among general population was observed in other countries, such as 16.9% in Netherlands (van der Velden et al., 2020), 27.8% in America (Ettman et al., 2020), 68% in Brazil (Goularte et al., 2020), and 41% in Spain (Rodríguez-Rey et al., 2020). This discrepancy in terms of prevalence of depression may be explained, to some extent, by differences in diagnostic criteria, severity of the epidemic, and intervention measures taken. The Chinese government had taken several measures to control and reduce COVID-19 transmission, such as city closure, traffic control, isolation at home, and wearing of masks. Majority of the Chinese general population considered that these measures were effective and believed that they would win the battle against COVID-19.

Our study found that younger individuals are more likely to have depressive symptoms compared with older individuals; similar results can be seen in the study of Huang and Zhao (2020), which concluded that younger people had a significantly higher prevalence of depressive symptoms than older people. However, most cross-sectional studies showed that higher age was associated with a higher risk of depressive symptoms in a non-disaster situation (Liu et al., 2015; Zhang et al., 2019). This discrepancy could be due to adaptive mechanisms, older people developed this ability to dealing with crises, which can be used to manage the stress associated with the current pandemic (Goularte et al., 2020). Additionally, young people tend to quickly obtain COVID-19 information from social media, which can easily trigger stress (Qiu et al., 2020).

The present study also showed that a higher prevalence of depressive symptoms was observed among individuals with a smoking habit, poor self-rated health, and low social support, which is consistent with most published studies (Pasco et al., 2008; Hu et al., 2018). Interestingly, depressive symptoms and the risk factors mentioned above had a bidirectional correlation. A 10 years study by Naicker et al. (2013) reported that depressed adolescents had higher proportion of smoking habit, poorer self-rated health, and lower social support compared with non-depressed adolescents 10 years later. Similarly, smoking habit, poor self-rated health, and low social support could increase the risk of depressive symptoms (Rousou et al., 2016; Holden et al., 2019).

Our study showed that individuals with an unclear understanding of the main symptoms of COVID-19 were more likely to report depressive symptoms, which is similar to the study of Wang et al. (2020b). The possible explanation for this phenomenon is that a more comprehensive understanding of the related knowledge about COVID-19 will help individuals face the epidemic rationally and judge effectively whether one is infected or not. Additionally, with a higher cognitive level regarding COVID-19, individuals would have more confidence and motivation to adopt effective measures to protect themselves. Therefore, improving public awareness of COVID-19 is necessary to improve mental health.

Our study suggested that staying in Wuhan within 3 months before the COVID-19 outbreak was associated with a higher risk of depressive symptoms. Lai et al. (2020) investigated 1,257 medical staff from fever clinics or wards of hospitals for patients with COVID-19 in China and reported that health-care workers in Wuhan had severe symptoms of depression, anxiety, insomnia, and distress. Wuhan is the origin and epicenter of the COVID-19 epidemic in China. Nearly 60% of the cases occurred in China, and a fatality rate of 5% was reported in Wuhan (Wu et al., 2020). Therefore, individuals with exposure in Wuhan before the COVID-19 outbreak were at an especially high risk for infection, which further increased the level of negative emotion, such as depression, anxiety, stress, and fear.

In this study, no significant correlation was observed between the number of local confirmed cases, COVID-19 infection status, COVID-19 latent period, route of transmission, and depressive symptoms. However, related studies have found that these factors may also affect the occurrence of depressive symptoms (Nelson et al., 2020; Zhang et al., 2020). This difference may be related to the study population and sampling methods. With the wide dissemination of information in China, the difference in the prevalence of depressive symptoms between severe and mild areas of the epidemic may have been weakened. Additionally, only 17 people reported that they or their relatives had COVID-19 infection in this study, which may affect the reliability of the results. Therefore, it is necessary to explore this correlation in a large sample population.

This study had multiple strengths. Firstly, the study included a large number of subjects, which made it possible to obtain more convincing results. Secondly, this study explored the association between factors connecting COVID-19 epidemic to depressive symptoms.

However, there were limitations too. First, we adopted a snowball sampling strategy. The snowball sampling selects potential respondents based on existing respondents, which is not a random sampling method. The recruitment information of study was posted on the university website, resulting in most of the respondents coming from young students. Therefore, there was bias in the selection of participants in our study and the study’s sample population may not be a good representation of the actual pattern of the general population. Second, our study was cross-sectional; we could not infer causal relationship of risk factors and symptoms of depression. Therefore, a cohort study is needed to certify this temporal relationship. Third, the CES-D is just a screening tool and not a diagnostic one, although it has been widely used and validated in China. Fourth, this study adopted a self-administered online questionnaire, thus participants need to be able to use network tools, which might have affected how they responded to the questionnaire. However, given the COVID-19 epidemic, an online survey was considered more appropriate.

## Conclusion

This study indicates that during the COVID-19 epidemic in China, a considerable proportion of the general population suffered from depressive symptoms. Those with younger age, a smoking habit, poorer self-rated health, and lower social support had a higher risk of depressive symptoms. Moreover, depressive symptoms were found to be significantly associated with knowledge about the main symptoms of COVID-19 and staying in Wuhan within 3 months before the COVID-19 outbreak. Given our findings, we recommend the establishment of targeted psychological interventions for the public to improve their mental health during the COVID-19 epidemic.

## Data Availability Statement

The original contributions presented in the study are included in the article/supplementary material, further inquiries can be directed to the corresponding author/s.

## Ethics Statement

The studies involving human participants were reviewed and approved by the Institutional Review Board of Tongji Medical College, Huazhong University of Science and Technology. The patients/participants provided their written informed consent to participate in this study.

## Author Contributions

SP participated all preparation of this manuscript. XL contributed to statistical analysis. YD and YL took part in the sample collection. KT and YG contributed to study design and the critical revision of the article. All authors reviewed the manuscript, approved the final draft, and contributed significantly to this work.

## Conflict of Interest

The authors declare that the research was conducted in the absence of any commercial or financial relationships that could be construed as a potential conflict of interest.
